# Etude de l’intérêt de l’éosinophilie sanguine au cours des exacerbations aiguës sévères de bronchopneumopathie chronique obstructive (BPCO) dans un centre tunisien

**DOI:** 10.11604/pamj.2019.34.138.17392

**Published:** 2019-11-08

**Authors:** Ahmed Ben Saad, Rim Khemakhem, Saousen Cheikh Mhamed, Nesrine Fahem, Asma Migaou, Samah Joobeur, Naceur Rouatbi

**Affiliations:** 1 Service de Pneumologie et d’Allergologie, Hôpital Universitaire Fattouma Bourguiba, Rue 1^er^ juin, 5000 Monastir, Tunisie

**Keywords:** Bronchopneumopathie chronique obstructive (BPCO), éosinophilie, exacerbation de la maladie, hospitalisation, phénotype, pronostic, Chronic obstructive pulmonary disease (COPD), eosinophilia, exacerbation of the disease, hospitalization, phenotype, prognosis

## Abstract

**Introduction:**

Il existe une controverse concernant la relation entre le taux d'éosinophiles sanguins (Eos) et la sévérité des exacerbations de BPCO. L'objectif de notre travail est de déterminer la relation entre le taux d'Eos et les différents paramètres de sévérité d'une exacerbation aiguë (EA) sévère de BPCO.

**Méthodes:**

Étude rétrospective monocentrique portant sur les patients porteurs de BPCO suivis entre 2005 et 2015 ayant nécessité l'hospitalisation. Nous avons comparé 2 groupes de patients: G1(Eos+): Eos ≥ 200 cell/μl (103 cas, 20,4%), G2(Eos-): Eos < 200 cell/μl (403 patients: 79,6%).

**Résultats:**

Cinq cent six patients BPCO ont été inclus dans l'étude. Il n'y avait pas de différence significative entre les 2 groupes concernant l'âge, le genre, le VEMS, et le nombre d'EA/an (Eos+:2.6, Eos-:2.5 EA/an; p = 0,48). L'analyse des paramètres de sévérité des EA sévères montre l'absence de différence entre les 2 groupes concernant la PaO2 mesurée à l'admission (60,5, 59,2 mmHg; p = 0,26), la capnie (p = 0,57), le pH (p = 0,74), le taux de CRP (mg/l) (82,7, 81; p = 0,89), la leucocytose (p = 0,36), le recours à la ventilation non invasive (5.8%, 6.5%; p = 0,81), la ventilation mécanique invasive (p = 0,5), la durée d'hospitalisation (9.7, 9 jours; p = 0,21), ainsi que le délai de la prochaine EABPCO (p = 0,32). La survie à 1 an était comparable entre les 2 groupes (94% vs 96%; Log Rank: 0,708).

**Conclusion:**

I'augmentation du taux d'Eos chez les patients BPCO ne semble pas avoir une influence péjorative au cours des EA sévères. Malgré la prise en considération du taux d'Eos dans les décisions thérapeutiques dans les récentes recommandations, l'importance et l'intérêt pronostique de l'Eos au cours de la BPCO pourrait être population dépendant.

## Abstract (en)

**Introduction:**

There is a controversy over the relationship between blood eosinophil count (BEC) and the severity of chronic obstructive pulmonary disease (COPD) exacerbations. The purpose of our study was to determine the relationship between blood eosinophil count and multiple parameters in assessing the severity of serious acute exacerbation (AE) of obstructive pulmonary disease.

**Methods:**

We conducted a retrospective monocentric study of hospitalized patients with obstructive pulmonary disease treated between 2005 and 2015. We compared 2 groups of patients: G1(BEC+): BEC ≥ 200 cell/μl (103 cases, 20.4%), G2(BEC-): BEC < 200 cell/μl (403 patients: 79.6%).

**Results:**

A total of 506 subjects with obstructive pulmonary disease were included in the study. No significant difference between the two groups in age, gender, forced expiratory volume in one second (FEV1), and the number of AE/year (BEC+: 2.6, BEC-:2.5 AE/year; p = 0.48) was found. The analysis of the parameters of severity of serious AE showed no difference between the two groups in partial pressure of oxygen PaO2 measured on admission (60.5, 59.2 mmHg; p = 0.26), capnia (p=0.57), pH (p=0.74), C-reactive protein rate (mg/L) (82.7, 81; p = 0.89), leukocytosis (p = 0.36), non-invasive mechanical ventilation (5.8%, 6.5%; p = 0.81), invasive mechanical ventilation (p = 0.5),length of stay in hospital (9.7, 9 days; p = 0.21), mean time to next AECOPD (p = 0.32). Survival at 1 year was comparable between the two groups (94% vs 96%; Log Rank: 0.708).

**Conclusion:**

Increased BEC in patients with COPD does not appear to have a negative effect on patients with severe AE. Despite the recent guidelines recommend to consider blood eosinophil count while making treatment decisions, the role and the prognostic interest of blood eosinophil count in patients with COPD could be population-dependent.

## Introduction

La bronchopneumopathie chronique obstructive (BPCO) est un problème majeur de santé publique. Elle représentera en 2020 la 3^ème^ cause de décès dans le monde [1]. C'est une maladie qu'on peut prévenir et traiter caractérisée par des symptômes respiratoires persistants et une limitation des débits aériens dues à des anomalies des voies aériennes et/ou des alvéoles souvent causées par une exposition significative à des particules ou gaz toxiques [2]. L'exacerbation aiguë de BPCO (EABPCO) est définie comme une aggravation aiguë des symptômes respiratoires nécessitant un traitement supplémentaire [1, 3]. Elle est associée à une augmentation de la mortalité et de la morbidité, et peut avoir un impact péjoratif sur la qualité de vie des patients [3, 4]. Cependant, les EABPCO sont assez hétérogènes. Plusieurs études ont été conduites afin de mieux comprendre l'hétérogénéité des patients ayant une BPCO, et d'identifier les différents phénotypes. Plus particulièrement, différents biomarqueurs ont été étudiés et constituent une voie de recherche intéressante dans ce domaine. Certaines études suggèrent que l'éosinophilie sanguine ou dans les expectorations est associée à un phénotype particulier des patients BPCO. En effet, l'hyperéosinophilie des voies respiratoires connue caractéristique de l'asthme, est désormais un marqueur de l'inflammation dans la BPCO [5-7]. Le pourcentage d'éosinophiles dans le sang périphérique est un biomarqueur simple et sensible pouvant refléter l'éosinophilie bronchique [8, 9]. Le dosage des éosinophiles dans le sang périphérique chez les patients BPCO est réalisé soit à l'état stable soit lors d'une EA. Bafadhel *et al.* [8] ont classé les patients ayant une EABPCO en quatre groupes biologiques distincts. Le groupe avec une hyperéosinophilie constituait 28% de toutes les exacerbations [8]. Le taux d'éosinophile sanguine (Eos) a été considéré comme un facteur prédictif indépendant de mortalité chez des patients BPCO en EA compliquée de pneumonie [10, 11]. Par ailleurs, une meilleure évolution sous corticothérapie systémique a été observée chez des patients BPCO éosinophiliques en EA dans d'autres études [12-14]. L'objectif de ce travail est d'étudier l'hypothèse selon laquelle les éosinophiles dans le sang périphérique pourraient servir comme marqueur pronostique et phénotypique dans les EABPCO.

## Méthodes

Il s'agit d'une étude observationnelle, rétrospective, monocentrique, incluant des patients suivis pour BPCO au service de pneumologie et d'allergologie à l'hôpital universitaire Fattouma Bourguiba de Monastir. Cette étude a été menée sur une période de 10 ans allant de janvier 2005 à décembre 2015. Les sujets inclus dans notre étude sont des patients porteurs de BPCO ayant eu au moins une hospitalisation pour EA sévère de BPCO et un suivi d'au moins 1 an après l'exacerbation. Le diagnostic de BPCO a été retenu devant: une symptomatologie respiratoire évocatrice du diagnostic (dyspnée, toux et expectoration chronique), une exposition significative à des particules toxiques notamment un tabagisme ≥ 10 paquet-année, et la présence à la spirométrie réalisée à l'état stable d'un trouble ventilatoire obstructif non complètement réversible après bronchodilatateur avec un rapport volume expiré à la première seconde lors d'une expiration forcée (VEMS) sur capacité vitale forcée (CVF) en post-bronchodilatation inférieur à 0,7. Une exacerbation aiguë sévère de BPCO a été définie comme une exacerbation aiguë nécessitant l'hospitalisation. Seuls les patients ayant bénéficié d'une numération de la formule sanguine (NFS) complète réalisée durant les 12 premières heures de l'admission ont été retenus dans cette étude. Le nombre d'éosinophiles par millimètre cube (mm^3^) a été précisé pour chaque patient à partir des NFS sus-citées. On a pris en considération le nombre d'éosinophiles de la première hospitalisation pour EABPCO. Cette dernière pouvait être soit chez des patients déjà connus porteurs de la maladie ou inaugurale. Les patients ont été ainsi répartis en 2 groupes: patients éosinophiliques (Eos+): Eosinophiles ≥200cellules/μl; patients non éosinophiliques (Eos-): Eosinophiles < 200 cellules/μl. Les données ont été recueillies à partir des dossiers des patients. Les sujets chez qui on a suspecté un chevauchement asthme BPCO (Asthma COPD overlap: ACO) selon les recommandations GINA (Global Initiative For Asthma) [15] n'ont pas étaient inclus dans l'étude. Les patients ont été évalués sur le plan épidémiologique (l'âge, le sexe, le tabagisme, l'indice de masse corporelle (IMC)), clinique (le nombre moyen des exacerbations modérés et sévères/an durant la période du suivi, la dyspnée (modified Medical Research Council (mMRC)) [16], biologique (numération formule sanguine, gazométrie artérielle, dosage de protéine C réactive), thérapeutique (oxygénothérapie, ventilation mécanique invasive, ventilation mécanique non invasive (VNI), corticothérapie) et évolutif (recours à la VNI, durée de l'hospitalisation, délai de la prochaine EA, taux de mortalité). L'analyse statistique a été réalisée à l'aide du logiciel SPSS version 18 (Statistical Package for the Social Sciences). Nous avons calculé les fréquences pour les variables qualitatives. Nous avons aussi calculé les moyennes, les médianes, l'écart type pour les variables quantitatives. Nous avons utilisé le test de Khi2 ou le test de Fisher pour comparer les variables qualitatives, le test de Student pour comparer les variables quantitatives. La probabilité de survie a été étudiée par la méthode de Kaplan-Meier avec des comparaisons effectuées par les tests de log Rank et Breslow. Le seuil de signification statistique a été fixé à 5%.

## Résultats

**Figure 1:**
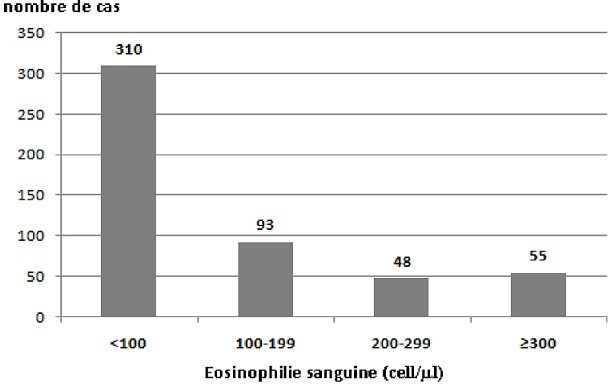
Répartition des patients BPCO en fonction du taux d’éosinophilie sanguine (éosinophilie sanguine: nombre/mm^3^)

Notre étude a inclus 506 cas parmi 715 patients hospitalisés durant la période d'étude. Quatre-vingt-dix-huit pourcent des patients étaient de sexe masculin (n = 496). L'âge moyen était de 68±10.3 ans et un VEMS moyen de 42.4±15.2% de la prédite. La plupart des patients étaient des fumeurs ou anciens fumeurs (98%) avec une médiane de paquets-années de 63.7 PA. En terme de GOLD (Global Initiative for Chronic Obstructive Lung Disease), 3% des patients étaient GOLD I, 28,9% GOLD II, 48,8% GOLD III, et 19,3% GOLD IV. Soixante et onze pourcents des patients étaient des “exacerbateurs fréquents”, ayant eu deux ou plusieurs exacerbations au cours des 12 derniers mois (Tableau 1). Cent trois patients (20,4%) avaient un taux d'éosinophiles ≥ 200 cell/μl (Groupe 1: Eos+) et 403 patients (79,6%) avaient un taux d'éosinophiles < 200 cell/μl (Groupe 2: Eos-) (Figure 1). Aucun des patients n'avaient des antécédents d'atopie. Les caractéristiques épidémiologiques (âge, genre, IMC), spirométriques (VEMS), le profil emphysémateux ou non au scanner thoracique, ainsi que la présence d'hypertension pulmonaire (HTP) à l'échocardiographie étaient comparables entre les deux groupes. Il n'y avait pas de différence significative entre les deux groupes concernant le nombre EA/an (Eos+: 2,6, Eos-:2.5; p = 0,48), le recours à l'oxygénothérapie au long cours (Eos+: 20,4% vs Eos-: 20,3%; p = 0,54), l'utilisation d'une ventilation non invasive à domicile (p = 0,12) ou l'utilisation de la corticothérapie inhalée (Eos+: 75,7%, Eos-: 67%; p = 0,07) ( Tableau 1). L'analyse des paramètres de sévérité des EA sévères montre l'absence de différence entre les 2 groupes concernant la PaO_2_ (pression artérielle en oxygène) mesurée à l'admission (60.5 versus 59.2 mmHg; p = 0,26), la capnie (p = 0,57), le pH (p = 0,74), le taux de la protéine C réactive (82,7 versus 81; p = 0,89) et la leucocytose (p = 0,36). Sur le plan thérapeutique, il n'y avait pas de différence significative concernant le recours à la VNI (5.8% versus 6.5%; p = 0,81), la ventilation mécanique invasive (p = 0,5) et la durée d'hospitalisation (9.7 versus 9 jours; p = 0.21). Une infection respiratoire basse à Pseudomonas aéruginosa et à Acinetobacter baumanii était présente dans 8,8% des patients Eos+ (versus 5,4% ; p = 0,22). Sur le plan évolutif, le délai de la prochaine EABPCO modérée ou sévère chez les patients Eos+ était de 190 jours (versus 220; p = 0,32). Les taux de prescription d'antibiothérapie et de corticothérapie systémique étaient similaires entre les deux groupes (Tableau 2). Il n'y avait pas de différence significative concernant le taux de mortalité intra-hospitalière entre les deux groupes (0% versus 1,2%; p = 0,31). L'étude de la survie n'a pas objectivé une différence significative entre les deux groupes. La survie à un an et à 3 ans était respectivement de 94% et 86% chez les patients Eos+ et 96% - 86% chez les patients Eos-(Log Rank: 0,708, Breslow: 0,744).

**Tableau 1:** Caractéristiques épidémiologiques et cliniques de la population étudiée

Variables	Tous les patients (n=506)	Groupe 1 (Eos+) (n=103)	Groupe 2 (Eos-) (n=403)	Valeur de p
**Age (en année)**	68±10.3	67.3±10,3	68,2±10,3	0,51
**Sexe (masculin)**	98 % (496)	100%	97,5%	0,1
**IMC (kg/m^2^)**	23,7±5,6	23,9±5,6	23,7±5,6	0,30
**VEMS (%)**	42,4±15,2	42.38%	42,5%	0,95
**VEMS(L)**	1,15	1.16	1,15	0,8
**Tabagisme (PA)**	63,7±29,5	64.8±30.9	63,46±30	0,67
**Exacerbateurs fréquents**	71,1%	73.8	70,5	0,29
**Groupe de GOLD (%)**				
I	3	2.9	3	0,63
II	28,9	31.1	28,2	0,32
III	48,8	42.7	50,4	0,10
IV	19,3	23.3	18,4	0,16
**Nombre EA/an**	2.52	2.62	2,5	0,48
**Utilisation des CSI (%)**	73,9	75,7	67	0,07
**Présence de HTP (%)**	13,6	12,6	13,9	0,87
**Profil emphysémateux (%)**	22,9	27,2	21,8	0,29
**IRC (%)**	58,3	60,2	57,8	0,73
**OLD (%)**	20,4	20,4	20,3	0,54
**VNI à domicile (%)**	2	3,9	1,5	0,12

**Abréviations:** VEMS : volume expiré à la première seconde lors d’une expiration forcée. IMC: l'indice de masse corporelle. CSI: corticostéroïdes inhalés. HTP: hypertension pulmonaire. IRC: insuffisance respiratoire chronique. OLD: oxygénothérapie de longue durée. VNI: Ventilation non invasive.

**Tableau 2:** Tableau comparatif des caractéristiques des exacerbations sévères entre les deux groupes

Variables	Groupe 1 (Eos+) (n=103)	Groupe 2 (Eos-) (n=403)	Valeur de p
PaO2 (mmHg)	60,5	59,2	0,26
PaCO2 (mmHg)	42,5	41,9	0,57
PH	7,39	7,39	0,74
CRP (mg/L)	82,7	81	0,89
Leucocytose (10^3^/μL)	12,8	12,6	0,36
VNI (%)	5,8	6,5	0,81
VMI (%)	1,9	1,5	0,5
Antibiothérapie (%)	86,4	87,1	0,85
Corticothérapie systémique (%)	71,8	73	0,82
Durée d’hospitalisation (jours)	9,7	9	0,21
Transfert en réanimation (%)	2,9	3,7	0,48
Délai de la prochaine EABPCO (jours)	190	220	0,32

**Abréviations:** CRP: protéine C réactive. VNI: Ventilation non invasive. VMI: Ventilation mécanique invasive

## Discussion

Notre étude visait à rechercher d'éventuelles relations entre le taux d'éosinophilie sanguine et la sévérité d'EABPCO. Un taux d'éosinophilie sanguine ≥ 200 cellules/μl mesuré lors d'une exacerbation sévère n'était pas associé dans ce travail à différents paramètres de sévérités biologiques, gazométriques, et évolutives. En effet, l'augmentation du taux d'Eos chez les patients BPCO ne semble pas avoir une influence péjorative au cours des EA sévères selon les résultats de notre étude. Malgré la taille de l'échantillon de notre étude, la disponibilité des données ayant permis d'apprécier la sévérité des EA et les suites évolutives, la comparabilité des deux groupes en termes de données démographiques, fonction respiratoire, traitement par CSI, risque d'EA, et prescription de corticothérapie systémique au cours des EA, notre étude présente quelques limites. D'une part, le nombre de patients inclus dans le groupe 2 (Eos-) était 3 fois plus important malgré un seuil à 200 cell/μl et nous n'avons pas étudié d'autres Cut off (300-400 cell/μl) où la taille de l'échantillon Eos+ serait réduite davantage. Néanmoins, les patients des deux groupes ne diffèrent pas en termes de caractéristiques cliniques, biologiques et fonctionnelles respiratoires. D'autre part, malgré l'absence de comptage des éosinophiles en pourcentage avec différents seuils, il est peu probable qu'ils auraient fourni des résultats différents dans notre étude. Nous avons pris en considération les patients dont la numération formule sanguine complète était réalisée durant les 12 premières heures de l'admission. Vu que notre étude est rétrospective, nous ne pouvons pas affirmer que tous les patients n'ont pas reçu une corticothérapie systémique préalable au prélèvement de la NFS à l'admission. Concernant les caractéristiques de la population étudiée, la plupart des patients inclus dans cette étude avaient une obstruction bronchique sévère à très sévère (GOLD III: 48.8%; GOLD IV: 19,4%) avec un nombre important de fréquents exacerbateurs. Ceci pourrait expliquer que 73,9% de nos patients soient sous corticothérapie inhalée. Ces constatations peuvent être dues au fait de n'inclure que des malades hospitalisés pour EABPCO. Quant à la stabilité ou la variabilité du l'Eos à travers le temps, les données de la littérature sont limitées et controversées [17]. Nous ne disposons souvent que d'un chiffre d'Eos lors des EABPCO pour notre population.

Certains travaux relatifs à la BPCO et éosinophilie ont étudiés la relation entre le taux d'Eos à l'état stable et le risque d'EA ainsi que la réponse à la corticothérapie inhalée (prise au long cours) en fonction du taux d'Eos. Nous ne disposons pas de mesure de l'Eos en dehors des EABPCO pour nos patients. Cependant, les 2 groupes sont comparables en termes de nombre d'EA/an, de VEMS ainsi que de traitement pas corticothérapie inhalée. Dans l'étude de Zysman *et al.* [18] incluant 458 patients atteints de BPCO, aucune différence significative concernant les symptômes, la fonction respiratoire, le taux d'EA et la survie à trois ans n'a été retrouvé entre les groupes de patients éosinophiliques et non éosinophiliques et cela pour différents seuils d'éosinophiles (2%, 3%, et 4%). Par ailleurs, selon Siva *et al.* [19], l'inflammation bronchique à éosinophiles est associée aux exacerbations de BPCO. De même, une réduction de polynucléaires éosinophiles dans l'expectoration est associée à une réduction des exacerbations de BPCO [19]. Vedel *et al.* [20], dans une cohorte de 203 patients suivis pour BPCO à Copenhague, ont trouvé qu'il y avait un taux plus élevé d'exacerbations sévères chez les patients ayant des taux Eos > 2% à l'état stable, alors que cette relation était inversée pour les exacerbations modérés. Pour certains, l'éosinophilie au cours de la BPCO constitue un biomarqueur associé au risque de survenue d'EA, de mortalité, de déclin de la fonction respiratoire ainsi qu'une meilleure réponse à la corticothérapie systémique ou inhalée [21]. Alors que dans la méta-analyse de Ho *et al.* [14], bien que les patients BPCO éosinophiliques ont une durée d'hospitalisation pour EA plus courte, une meilleure qualité de vie et une meilleure réponse à la corticothérapie inhalée par rapport au groupe de patients non éosinophiliques, ces deux groupes partagent le même risque d'EA/an et de mortalité. Cependant, les résultats d'une analyse combinée de l'étude SPIROMICS (Subpopulations and Intermediate Outcomes Measures in COPD Study) (n = 1,544) et COPDGene (Genetic Epidemiology of COPD) (n = 602), ont été décevants et ont conduit les auteurs à conclure que, bien que certains biomarqueurs sanguins étaient significativement associés à la survenue d'exacerbations, aucun n'était robuste entre les cohortes. En outre, les biomarqueurs, tous ensembles, ajoutent peu à la valeur prédictive des caractéristiques cliniques des EABPCO [22].

La relation entre le taux d'Eos mesuré à l'admission au cours des EA sévères de BPCO et les particularités évolutives de l'épisode reste conflictuelle. Nous n'avons pas retrouvé de relation entre ce taux et la sévérité de l'épisode notamment en termes de données gazométriques, évolutives, durée du séjour hospitalier ou délai de la prochaine EABPCO. Dans un essai multicentrique randomisé contrôlé mené par Bafadhel *et al.* [23], ayant inclus 243 patients BPCO hospitalisés pour EABPCO, 25% des cas avaient un taux d'Eos mesurée à l'admission ≥ 200 cells/μL et/ou ≥ 2%. Les patients présentant des EA éosinophiliques avaient une durée d'hospitalisation plus courte par rapport au groupe de patients non éosinophliques avec un taux de réadmission à 12 mois comparable entre les deux groupes. La comparaison de la durée du séjour hospitalier montre une différence statistiquement non significative entre les 2 groupes en excluant les cas avec pneumonie. Ce qui rejoint nos résultats. Une autre étude faite en Turquie où un plus faible risque de réadmission et un séjour hospitalier plus court ont été démontrés dans le groupe BPCO avec un taux d'éosinophiles à l'admission élevé [24]. Cette évolution favorable a été retrouvé même en cas d'hospitalisation en unité de soins intensives avec une durée d'hospitalisation plus courte et un taux de mortalité plus bas [25]. Des résultats différents ont été retrouvés dans le travail de Couillard *et al.* où le risque de réadmission à 12 mois était plus élevé pour les patients BPCO avec une éosinophilie sanguine à l'admission ≥ 200 cells/μL et/ou ≥ 2% [26]. Kolsum *et al.* [27] ont trouvé une relation inverse entre l'infection bactérienne et les polynucléaires éosinophiles dans le sang chez les patients atteints de BPCO. Ils ont trouvé que les patients présentant une EA infectieuse bactérienne, avaient une diminution significative de la numération absolue des éosinophiles dans le sang par rapport à l'état stable. Ceci pourrait expliquer l'absence de relation entre le taux d'Eos mesuré lors des EABPCO et la sévérité de ces épisodes. Il n'y avait pas de différence significative des taux de mortalité entre les 2 groupes dans notre travail. Plusieurs travaux ont montré que l'éosinophilie sanguine peut être associée à une augmentation de la mortalité toutes causes confondues chez les sujets ayant une maladie respiratoire chronique par rapport aux sujets sains [28, 29]. Dans l'étude de Bafadhel *et al.* [23], les taux de mortalité à 12 mois étaient similaires entre les deux groupes éosinophiliques et non éosinophiliques.

Toutefois, certaines questions restent à poser. Par exemple, il n'y a pas de consensus au sujet de l'utilisation de valeur absolue ou relative de polynucléaires éosinophiles, ni sur le taux d'éosinophiles circulants recommandé pour de meilleurs résultats comparatifs. Ainsi différents seuils sont appliqués dans les travaux rendant la littérature hétérogène [18, 30, 31]. Les données de la littérature concernant la variabilité intra-individuelle du taux d'Eos chez les patients BPCO au cours du temps sont limitées. En effet, il n'est pas possible actuellement d'affirmer que la présence d'une hyperéosinophilie à l'état stable soit corrélée à un taux élevé d'Eos lors des EA et inversement. Concernant la discordance des données de notre étude et d'autres en termes de corrélation entre Eos et l'aggravation du risque lors des EA sévères de BPCO, l'exploration de la valeur prédictive d'autres biomarqueurs parait nécessaire. D'autres études ciblant les EABPCO éosinophiliques sont nécessaires pour une meilleure appréciation du pronostic et des choix thérapeutiques [32-34].

## Conclusion

La principale conclusion tirée de cette étude est l'absence de différence significative dans le pronostic et dans la plupart des caractéristiques cliniques, biologiques et fonctionnelles entre les patients en exacerbation sévère de BPCO ayant des taux d'éosinophilie sanguine supérieurs ou inférieurs à 200 cell/μl. Malgré la prise en considération du taux d'Eos dans les décisions thérapeutiques dans les récentes recommandations, l'importance et l'intérêt pronostique de l'Eos au cours de la BPCO pourrait être population dépendante. Ceci témoigne de l'hétérogénéité de la maladie et que le phénotypage des patients BPCO doit tenir compte de différentes variables constitutionnels et environnementales. D'autres études sont nécessaires pour une meilleure appréciation de ce biomarqueur de phénotypage des patients BPCO.

### Etat des connaissances actuelles sur le sujet

- Les éosinophiles dans le sang périphérique pourraient servir comme marqueur biologique pronostique et phénotypique dans les exacerbations aiguës de BPCO;
- Il existe une controverse concernant la relation entre le taux d'éosinophiles sanguins et la sévérité des exacerbations de BPCO.

### Contribution de notre étude à la connaissance

- A notre connaissance, ce travail constitue l'un des premiers travaux à s'intéresser à la relation entre l'éosinophilie sanguine et la sévérité des exacerbations aiguës de BPCO dans une population africaine;
- L'augmentation du taux d'Eos (≥ 200 cellules/μl) chez les patients BPCO ne semble pas avoir une influence péjorative au cours des EA sévères; l'intérêt pronostique de l'Eos au cours de la BPCO pourrait être population dépendante;
- Ce travail conforte l'aspect conflictuel que porte l'étude de l'éosinophilie sanguine au cours de la BPCO.

## Conflits d’intérêts

Les auteurs ne déclarent aucun conflit d'intérêts.
